# Complete Genome Sequence of PM105, a New *Pseudomonas aeruginosa* B3-Like Transposable Phage

**DOI:** 10.1128/genomeA.01543-15

**Published:** 2016-03-03

**Authors:** Christine Pourcel, Cédric Midoux, Maria Bourkaltseva, Elena Pleteneva, Victor Krylov

**Affiliations:** a Institute for Integrative Biology of the Cell, CNRS, CEA, Univ. Paris-Sud, Université Paris-Saclay, Orsay, France; b I. I. Mechnikov Research Institute for Vaccines & Sera, Moscow, Russian Federation

## Abstract

The complete genome of the *Pseudomonas aeruginosa* bacteriophage PM105 is 39,593 bp long. The phage belongs to the B3 family of transposable Mu-like phages, as confirmed by the presence of bacterial DNA joined to the phage genome ends. PM105, together with other B3-like phages, form a newly arising species.

## GENOME ANNOUNCEMENT

Several *Pseudomonas aeruginosa* generalized transducing phages, showing similarity with *Escherichia coli* phage Mu, have been isolated (1). These temperate phages have the ability to replicate their genome through transposition into the bacterial chromosome as a step in their life cycle. Comparison of their genomes allowed the distribution of these phages into two subgroups: D3112-like phages (D3112 [2] and 15 other phages) and B3-like phages (including B3 [3] and HW12) (4).

Bacteriophage PM105 was isolated from a Moscow clinical isolate. The morphology of phage PM105 virion was determined using transmission electron microscopy (performed at 80 kV in JEM-100B with the use of negative contrast with 1% uranyl acetate) showing a 60-nm head and a 200-nm-long flexible tail, the characteristics of *Siphoviridae*. Phage DNA was sequenced in an Illumina MiSeq 250-bp paired-end run, with a 250-bp insert library at the IMAGIF sequencing facility. Quality-controlled trimmed reads were assembled, using Geneious, into a single linear contig at a mean coverage of 15,287-fold. The PM105 genome is 39,593 bp long, with a G+C content of 63%. The presence of 600-bp to 2-kb bacterial DNA fragments on the right part of its genome, a characteristic of Mu-like phages, suggests that it replicates through transposition. The PM105 genome sequence reveals 58% and 77% matches with 91% and 97% identity to the B3 (accession no. AF232233) and JBD 18 (accession no. JX495041) genomes, respectively (5). The gene arrangement is similar to that of B3-like phages, with some differences. There are genes in several regions that appear to be completely different in B3 and PM105, and their function is not known. Such regions, found also in genomes of other B3-like phages (JBD18, JBD67, and JBD25), might correspond to genome gaps filled with random nucleotide sequences with a structural function, or to genes of different origins essential for phage viability. Phage B3 open reading frame 48 (ORF48), encoding a methylase, is not found in any other B3-like phages, including PM105. Using Prodigal (6), ribosome binding sites (RBS) were predicted 5- to 10-bp upstream of all the annotated genes, with the exception of ORF46, where a putative RBS was seen at +13 nucleotides to the ATG start codon.

All D3112-like phages are considered to be one species (with >70% genome identity), whereas a comparison of B3, PM105, and HW12 shows that their genome identity is <70% (4). However, these can be grouped into a new species on the basis of their gene organization. The three phages are heteroimmune. PM105, unlike other transposable phages, exhibits sensitivity to incubation at 42°C. Similarly to other B3-like phages, and in contrast to D3112-like phages, it integrates upon lysogenization at random sites in the bacterial chromosome, inducing auxotrophic or other mutations. In the course of lytic development, there are differences for В3 and D3112 in the choice of sites for integration, as shown by sequencing of the bacterial DNA attached to one end of the phage genome.

### Nucleotide sequence accession number.

The complete sequence of *P. aeruginosa* phage PM105 has been deposited in European Nucleotide Archive (ENA) under the accession no. LN898172. The version described in this paper is the first version.
